# ChatGPT Relies More Heavily on Consonants Than on Vowels to Recognize Words

**DOI:** 10.5334/joc.487

**Published:** 2026-02-09

**Authors:** Juan Manuel Toro

**Affiliations:** 1 Institució Catalana de Recerca i Estudis Avançats (ICREA), Barcelona, Spain; 2 Universitat Pompeu Fabra, Barcelona, Spain

**Keywords:** Consonant bias, Word recognition, LLMs, Artificial Intelligence, Statistical learning

## Abstract

Humans develop biases during language learning. For example, we rely more heavily on consonants than on vowels to identify words. Advances on artificial intelligence have allowed the development of proficient large language models that sometimes mimic humans’ language use. They do so by tracking regularities in natural language datasets that are used to train them. Here we test the hypothesis that tracking such regularities is enough for the emergence of responses that resemble the consonant bias. We asked ChatGPT which of two nonsense words (one with a vowel and one with a consonant change) was more similar to a target word. We observed that the model uses more the consonants than the vowels to perform similarity judgments across words in the two languages that we tested (English and Spanish).

## 1. Introduction

In humans, acquiring a language leads to the emergence of processing biases. A well-known example is that of the consonant bias. This consists in a strong reliance on consonants over vowels for lexical access. However, the origins of this bias are still unknown. Here, we take advantage of advances in artificial intelligence to explore the conditions that allow for its emergence. In recent years, large language models (LLMs) have become increasingly proficient at producing not only short answers to an users’ questions, but also increasingly complex texts that are difficult to differentiate from college-level essays. To acquire such levels of proficiency, LLMs are trained with extensive databases of written language taken from the internet. In the present experiment, we test the hypothesis that such training with natural language is enough for a learner (in this case, an artificial learner) to develop a focus on consonants to identify words. The observation of such bias in the model could provide information about the minimal conditions that might support its emergence in humans. Additionally, it will offer insights about the capabilities of current artificial language learners.

The consonant bias has been observed across different native languages (including English [van Ooijen, 1996], French [New, Araujo & Nazzi, 2008], Spanish [Duñabeitia & Carreiras, 2011], Dutch [Cutler, Sebastian-Gallés, Soler-Vilageliu & van Ooijen, 2000], Arabic [Boudelaa, 2015] and Hebrew [2025]), different perceptual modalities (oral [Cutler, Sebastian-Gallés, Soler-Vilageliu, & van Ooijen, 2000], written [New, Araujo & Nazzi, 2008]), and different experimental tasks (including word learning [Nazzi, 2005], word recognition [Bouchon, Hochmann & Toro, 2022], word reconstruction [Cutler et al. 2000], masked priming [Perea & Lupker, 2004] and speech segmentation [Bonatti, Peña, Nespor & Mehler, 2005]). More relevant for the present study is the consistent evidence showing a focus on consonants to recognize written words. In a masked priming task, primes that keep the consonantal tier of the target word prime as much as primes identical of the target word. In contrast, primes that keep the vowel tier of the target word do not produce a reliable priming effect (New, Araujo & Nazzi, 2008; Soares, Perea & Comesaña, 2014). A similar effect is observed when using a relative position priming paradigm in which either only the vowels or only the consonants of a word are used as primes (Duñabeitia & Carreiras, 2011). In fact, primes composed of only consonants produce similar neural electrophysiological responses to those of identical primes, while primes composed only of vowels do not (Carreiras, Duñabeitia & Molinaro, 2009). Delaying the visual presentation of the consonants composing a word generates an earlier neural response (linked to the identification of the word) than the presentation of the vowels composing the same word (Carreiras, Gillon-Dowens, Vergara & Perea, 2008). Even more, studies have observed different brain activation patterns during a reading and a written word recognition task when the available information comes from consonants or from vowels (Carreiras & Price, 2008; see also Caramazza, Chialant, Capasso & Miceli, 2000), There is thus consistent evidence that humans have a strong bias that favors the use of consonants for the identification of written words.

Do current LLMs display a similar focus on consonants to identify words? To effectively generate convincing texts, LLMs are trained on increasingly larger sets of linguistic stimuli. Through training, the models detect regularities between the elements in the corpus and learn to predict what element most likely follows another in a given context. Importantly, no a priori preference for consonants or vowels is built into the neural networks that support LLMs (e.g. Brown et al., 2020). So, the rise in sophistication of these models opens the door to test predictions about language processing in the absence of prior representations. Natural languages tend to have more consonants than vowels. Maddieson (2013) reported that all of the 563 languages that he analyzed had more consonants than vowels. Thus, listeners exposed to this asymmetry between consonants and vowels might learn to rely more on consonants to access the lexicon, as they are more informative to disambiguate between different words (Keidel, Jenison, Kluender & Seidenberg, 2007; see also Bonatti, Peña, Nespor & Mehler, 2007). Thus, the hypothesis we test here is that training with natural language input is enough for the LLMs to display responses that resemble the focus on consonants that has been observed in humans.

## 2. Methods

For the present study, we prompted a publicly-available LLM (OpenAI’s ChatGPT, model GPT4o; all data was collected from June to July, 2024; we used the default settings in ChatGPT and no additional settings or features were enabled) to compare two foils to a target word. More specifically, we asked the model which of two non-words, one with a vowel change and one with a consonant change, was more similar to a target word. We used the prompt “If you have to choose, which of these two words is more similar to [target word], [non-word 1] or [non-word 2]?” (e.g. “If you have to choose, which of these two non-words is more similar to “natural”, “nateral” or “nalural”?”; see Table 1 for examples of target words and non-words). As targets, we selected 100 words in English (for a complete list of words used as targets and non-words, please see accompanying supplementary materials). Crucially, the words had different length (2, 3 and 4 syllables), syllabic structure (CV, CVC, CCV and VC) and belonged to different syntactic categories (nouns, verbs and adjectives). To create the non-words, we changed either 1 vowel or 1 consonant in each target word. The change could be placed in any syllable of the target word. However, we tried to avoid making changes in the first syllable of words (we only changed the first syllable in 1 Spanish and in 2 English words). Across different words, the changes were implemented in the onset, nucleus or coda of syllables. Importantly, the 2 non-words presented in each trial had their vowel and consonant change implemented in the same syllable. That is, for a given target word, both non-words would have either the consonant or the vowel changed in the second syllable, third or fourth syllable (see for example Table 1), so distance and structure are balanced across consonant and vowel conditions. The order of presentation of the non-words within the prompt was balanced across trials. So, in half of the trials the non-word with a consonant change was presented first and in the other half of the trials the non-word with a vowel change was presented first. ChatGPT learns from previous interactions with the user, so no feedback was provided after its responses. Also, prompts were introduced one by one, in a new chat window for each prompt, so no automated script was used for their presentation.

**Table 1:** Examples of English target words and non-words. Non-words had either one consonant or one vowel change.


TARGET	C CHANGE	V CHANGE

astronaut	asgronaut	astrinaut

chocolate	chocorate	chocolite

elegant	elegart	elegunt

father	fatheg	fathor

imaginative	imapinative	imagunative

magic	magil	magec

obsolete	obdolete	obsilete

river	rives	rivur

understand	undepstand	undorstand

zebra	zetra	zebru


We also queried the model in Spanish. The prompt was “Si tienes que elegir, cual de estas dos no-palabras se parece más a [target word], [non-word 1] o [non-word 2]?”. We selected as targets 100 words in Spanish (see accompanying supplementary materials). The creation and presentation of the non-words was the same as in English. To control for orthographic plausibility of the non-words that we created, we measured bigram frequency in the target words and the corresponding consonant and vowel non-words in both English and Spanish. An ANOVA over bigram frequency and the variables Language (English, Spanish) and Item (Target, Consonant change, Vowel change) showed no differences between the target and the non-sense words (F(2, 1176) = 0.226, p = 0.795), no differences between English and Spanish (F(1, 1176) = 2.264, p = 0.133) and no interaction between them (F(2, 1176) = 0.055, p = 0.946). Thus, the non-words that we used did not differ from the actual target words in how plausible they were from an orthographic point of view, and more importantly, there were no orthographic differences between the consonant and the vowel non-sense words.

To be sure that any observed effects were not due to any idiosyncratic feature of a specific ChatGPT account, we ran the experiment (both the English and the Spanish lists) using 22 different accounts. We thus recruited 22 different participants that created a ChatGPT account for themselves and provided them with the prompts and the list of words to be tested. We asked the participants to register the chatbot’s response for every trial in a response sheet in terms of whether it choose as more similar to the target word the foil with a consonant change or the foil with a vowel change. The participants were blind to the hypothesis being tested and received monetary compensation for their participation in the study.

## 3. Results

**Figure 1:**
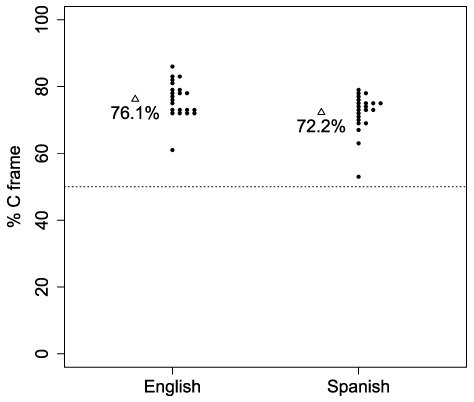
Mean percentage (and individual data points) of trials in which ChatGPT choose non-words that kept the consonantal frame over non-words that kept the vowel frame as more similar to a target word in both English and Spanish. Dotted line is chance level.

In English, ChatGPT chose the non-word that left the consonantal tier of the target word intact over the non-word that preserved the vowel tier in 76.1% (SD = 5.4) of the trials. Similar results were observed in Spanish (72.2%, SD = 5.7; see Figure 1). To analyze the responses observed across the 22 different runs in both English and Spanish, while taking into account the different sources of potential variability (e.g. length of the target word or different users), we used a generalized linear mixed model (using the gmler command from the lme4 package [v1.1.35.5; Bates, Mächler, Bolker & Walker, 2015] in R [v4.4.2; R Core Team, 2021]). In the model, the fixed effects were Language, Word length and Syllable position, while the random intercepts were User and Target word. None of the fixed effects were significant (Language: z = –0.93, p = 0.53, with a regression coefficient [in odds ratio] of 0.824; Word Length: z = –0.026, p = 0.978, regression coefficient = 0.995; Syllable Position: z = 0.112, p = 0.91, regression coefficient = 1.032). Importantly, the model showed that the effect that we observed (ChatGPT choosing non-words that left the consonantal tier intact) was highly independent of the variability present in the data (z = 2.782, p = 0.0054, regression coefficient = 5.823). Thus, the results suggest that ChatGPT relies more heavily on consonants than on vowels while performing similarity judgements across words.

## 4. Discussion

The original hypothesis about the consonant bias is that it facilitates language learning by establishing a “division of labor” between consonant and vowels (Nespor et al. 2002). While consonants mainly provide lexical information, vowels provide indexical information, and infants might benefit from this division of labor to discover the regularities in language (e.g. Hochmann, Benavides-Varela, Nespor & Mehler, 2011). In the present study, we observed that ChatGPT tends to judge as more similar to a target word a foil that preserves the consonants over one that preserves the vowels. Thus, a LLM that has been trained with natural linguistic data seems to use more frequently the consonants than the vowels to perform similarity judgements across words.

LLMs are based on complex neural networks that process and generate text based on statistical patterns learned from their training data. Most popular LLMs, like ChatGPT, are based on the transformer architecture. For character/word generation (depending on the token size), a trained LLM uses iteratively the statistics extracted in the training phase to assign probabilities for the following character of a sequence. For this, the model uses a byte pair encoding algorithm that allows for the tokenization of the input used to train it while keeping track of their frequency. Thus, following a previous context, the model is able to predict how likely is a given token (either a character or a word) to come. At the same time, it can measure how unlikely is a token sequence (for example, a character to be present in an unseen word), comparing its predictions with its “experience”. But the transformer architecture goes beyond mere statistical learning to produce predictions, as it uses an attention mechanism that weighs the relative importance of the different possible outcomes (words in a text generation task) and it is fine-tuned to produce text that more closely resembles human writing. It might thus be the case that, by tracking the combination of consonants and vowels present in a given natural language, LLMs learn to assign a higher predictive value to the consonants to disambiguate between possible lexical tokens.

Of course, the argument here is not that the model is learning to process written text similarly to how human infants acquire language (e.g. Chomsky, Roberts & Watumull, 2023; Marcus, 2018). What we find interesting from these results is that current LLMs that have been trained with extensive natural language datasets, seem to extract more information from the consonant segments than from the vowel segments while judging the similarity across words. One open question is the level at which such focus might be learned by the model, as it can emerge from tokenization and sub-word statistics or from orthographic distributional regularities. However, these results offer cues about the minimal conditions that might allow for the emergence of a consonant bias in humans (Nazzi et al., 2016). A learner tracking the dependencies present in the linguistic signal might progressively focus on the consonants to disambiguate words as they provide more information about distinct lexical items (e.g. Keidel et al., 2007).

In our results, we observed that the preference for non-words that preserved the consonant frame was present in the two languages that we tested. However, it was a bit higher in English than in Spanish. This very likely reflects the size of the training dataset presented to the LLM. For reference, up to 93% of the data used to train GPT-3 is in English, and only up to 0,8% of the data is in Spanish (Brown et al. 2020). Thus, the language model has received substantially more training in the former than in the latter, likely allowing it to make more accurate predictions of character sequences. But more importantly, this preference to use consonants over vowels while performing similarity judgements across words is not built into current large language models (Brown et al., 2020). It seems to be a property that emerges from the training that prepares these models to process language and their continuous use of it.

Finally, it should be noted that the task we implemented in the present study differs from classical experimental tasks that have been used to explore the consonant bias in humans in the written domain. For example, using a masked priming paradigm (e.g. Duñabeitia & Carreiras, 2011; New et al., 2008; Perea & Lupker, 2004; Soares et al., 2014) participants tend to respond faster in a lexical decision task when the primes share the consonant tier with the target than when the primes share the vowel tier. Similarly, different neural responses have been observed using both EEG (Carreiras et al., 2009) and fMRI (Carreiras et al. 2008; Carreiras & Price, 2008) techniques when the participants are presented with written targets in which either the consonants or the vowels of a target word are manipulated. While the experimental task that we use here is not a direct replication of those studies, it does involve making similarity judgments between a target word and foils that have a change in either a consonant or a vowel. Further experiments could explore the possibility of measuring a model’s reaction times to implement a masked priming paradigm. It would also be interested to test whether measuring the network’s internal patterns of activation might produce results that are similar to those observed in humans’ brains while reading consonant and vowel foils.

The level of sophistication with which current LLMs mimic language processing opens the door to the opportunity of testing the conditions that allow certain behaviors observed in humans to potentially emerge in the absence of prior representations. Conversely, this also poses the question of whether the implementation of biases found in natural cognitive systems might improve the performance of artificial intelligences by directing them towards relevant sources of information (see for example Toro, 2024). The present study suggests that both natural and artificial learners tracking the dependencies that are present in the linguistic signal will focus on the consonants as the most informative parts of the signal to assess the similarity across words.

## Data Accessibility Statement

All datasets generated for this study, together with all stimuli used, are available in the supplementary materials.

## Additional File

The additional file for this article can be found as follows:

10.5334/joc.487.s1 Supplementary material. Tables 1 to 3.
