# Supplementary material for: ChatGPT Relies More Heavily on Consonants Than on Vowels to Recognize Words
Source: J Cogn. 2026 Feb 9;9(1):15. doi: 10.5334/joc.487 (PMC12908626; doi:10.5334/joc.487)
Supplement: Supplementary material. — Tables 1 to 3. [file joc-9-1-487-s1.pdf]

**Supplementary material**

**ChatGPT relies more heavily on consonants than on vowels to recognize words**

**Table 1.** List of 100 target words and non-words used for the English prompt "If you have to choose, which of these two non-words is more similar to [target word], [non-word 1] or [non-word 2]?". Non-words had either one consonant or one vowel change.

| Target | C change | V change |
|---|---|---|
| acid | acif | aced |
| adventure | adfenture | advonture |
| ambitious | ambilious | ambitaous |
| amicable | amifable | amicoble |
| argument | arsument | argiment |
| astronaut | asgronaut | astrinaut |
| attitude | attilude | attitade |
| avocado | avolado | avocudo |
| baby | maby | buby |
| barbecue | barnecue | barbocue |
| beautiful | beausiful | beautoful |
| bicycle | binycle | bicacle |
| brilliant | brilliart | brilliunt |
| calculate | calgulate | calcelate |
| calendar | camendar | calondar |
| camera | calera | camura |
| category | catelory | categary |
| chocolate | chocorate | chocolite |
| clever | clefer | clevor |
| compassionate | comnassionate | compossionate |
| computer | comluter | compater |
| confident | conrident | confodent |
| construction | consprucion | constrection |
| conversation | condersation | convirsation |
| design | delign | desegn |
| dinosaur | dilosaur | dinesaur |
| disparity | disnarity | dispurity |
| diva | dina | divu |
| document | docufent | docament |
| economy | ecoromy | econimy |
| elegant | elegart | elegunt |
| elephant | emephant | elophant |
| energetic | enedgetic | enurgetic |
| even | evef | evun |
| exercise | exencise | exurcise |
| explore | explome | explora |
| father | fatheg | fathor |
| flower | flomer | flowir |
| formidable | formisable | formidoble |

| | | |
|---|---|---|
| generate | genesate | generote |
| generous | gederous | genarous |
| honest | honent | honust |
| hospital | hosmital | hosputal |
| imaginative | imapinative | imagunative |
| improve | imfrove | imprave |
| inevitable | inegitable | inevutable |
| infection | infeltion | infuction |
| insert | insent | insart |
| intelligent | inmelligent | intolligent |
| investigate | invesligate | investugate |
| island | islard | islond |
| keyboard | keymoard | keybeard |
| lemon | leton | lemin |
| library | ligrary | librory |
| listen | lisfen | listun |
| logical | lopical | logacal |
| magic | magil | magec |
| manage | mapage | manige |
| mansion | mansiod | mansian |
| metaphor | meraphor | metiphor |
| microphone | microsone | microphune |
| modest | modert | modost |
| mysterious | myslerious | mystorious |
| natural | nalural | nateral |
| notebook | nosebook | notubook |
| obsolete | obdolete | obsilete |
| open | opes | opun |
| optimistic | optimiltic | optimostic |
| organize | ordanize | orgunize |
| paper | pamer | papor |
| parent | parelt | parunt |
| passionate | passiodate | passionite |
| powerful | powenful | poworful |
| relax | relan | relix |
| river | rives | rivur |
| romantic | romaltic | romintic |
| satisfaction | satislaction | satisfoction |
| sensitive | sensipive | sensitove |
| sincere | sintere | sincure |
| social | sociam | sociul |
| study | sfudy | stidy |
| table | tabre | tabli |
| telephone | teledone | telephine |
| tomato | topato | tomuto |
| travel | traved | travul |
| triangle | triasgle | triengle |
| trustworthy | trustwopthy | trustwerthy |
| ultimate | ultisate | ultimote |
| umbrella | umfrella | umbrilla |

| | | |
|---|---|---|
| understand | undepstand | undorstand |
| universal | unitersal | univorsal |
| universe | upiverse | unaverse |
| vanilla | vasilla | vanulla |
| vegetable | vegemable | vegetible |
| victory | vicpory | victary |
| vivacious | vipacious | vivecious |
| volunteer | volulteer | volanteer |
| write | wrine | writu |
| yesterday | yestenday | yesturday |
| zebra | zetra | zebru |

**Table 2.** List of 100 target words and non-words used for the Spanish prompt “Si tienes que elegir, cual de estas dos no-palabras se parece más a [target word], [non-word 1] o [non-word 2]?”. Non-words had either one consonant or one vowel change.

| Target | C change | V change |
|---|---|---|
| abierto | abiento | abiorto |
| abrazo | acrazo | abrizo |
| aburrido | amurrido | aberrido |
| agua | alua | agia |
| alegre | adegre | aligre |
| alto | almo | alte |
| alud | alun | aled |
| animal | animad | animol |
| aprender | apresder | aprunder |
| árbol | árbon | árbil |
| arco | arfo | arcu |
| armario | arbario | armurio |
| balón | balós | balín |
| bocadillo | bonadillo | bocudillo |
| bolígrafo | bosígrafo | bolágrafo |
| bonito | bosito | bonuto |
| brillante | brillacte | brillente |
| caballo | calallo | cabillo |
| calabaza | calaraza | calabiza |
| calcetín | caldetín | calcitín |
| caldero | calmero | caldiro |
| calendario | calesdario | calondario |
| cálido | cáfido | cáludo |
| caliente | calieste | calionte |
| caminar | cabinar | camunar |
| carrusel | carrunel | carrusul |
| casa | cafa | casu |
| celebrar | cepebrar | celobrar |
| cerrado | cerrafo | cerradi |
| césped | césred | céspud |
| cocinar | cocisar | cocinur |

| | | |
|---|---|---|
| comer | coter | comor |
| comercio | comencio | comurcio |
| compartir | compastir | compurtir |
| complicado | complifado | complicudo |
| concentración | concenpración | concentrución |
| concierto | conciepto | conciurto |
| conectar | conentar | conictar |
| conservar | consenvar | consurvar |
| construir | constluir | constreir |
| decidir | detidir | decedir |
| delgado | delmado | delgodo |
| descubrir | desdubrir | descabrir |
| despertar | despentar | despurtar |
| difícil | dibícil | difécil |
| dirigir | diribir | dirigar |
| divertido | divestido | divurtido |
| dormida | dorpida | dormoda |
| enseñar | enkeñar | ensiñar |
| épico | énico | épuco |
| escribir | esclibir | escrobir |
| escritorio | escrilorio | escritario |
| escuchar | esnuchar | escochar |
| estudiar | estumiar | estuduar |
| filtro | filpro | filtri |
| flor | flon | flir |
| fuerte | fuerde | fuerti |
| grande | granfe | grandu |
| hablar | hablaf | hablir |
| hermoso | herdoso | hermeso |
| horrible | horribre | horriblu |
| hospital | hosfital | hospatal |
| insecto | inselto | insocto |
| instante | insfante | instunte |
| interesante | intenesante | interosante |
| investigar | invesfigar | investugar |
| invierno | inviesno | inviurno |
| iris | irin | ires |
| jirafa | jinafa | jirofa |
| joven | jovel | jovun |
| manzana | mandana | manzina |
| marmota | marlota | marmeta |
| mejora | mebora | mejera |
| nadar | nadap | nadur |
| nariz | narip | naruz |
| niña | nifa | niñu |
| observar | obselvar | obsirvar |
| oler | ober | olir |
| opaco | oñaco | opico |
| ordenador | orfenador | ordunador |
| pantalón | pandalón | pantilón |

| | | |
|---|---|---|
| pequeño | pequedo | pequeñe |
| primero | prifero | primuro |
| rápido | rálido | rápudo |
| reparar | relarar | reporar |
| satélite | samélite | satúlite |
| segundo | segurdo | segendo |
| sencillo | sentillo | sencello |
| sílaba | sígaba | síloba |
| sombrilla | somgrilla | sombrolla |
| soñar | sodar | soñir |
| tambor | tambos | tambar |
| trabajar | tradajar | trabojar |
| tranquilo | tranquipo | tranquile |
| triángulo | triásgulo | trióngulo |
| universo | univetso | univurso |
| valiente | valierte | valionte |
| visitar | vinitar | visatar |
| zapato | zadato | zapeto |

**Table 3.** Percentage of trials in which ChatGPT choose the word that kept the consonant frame as more similar to the target word across the 22 different users in both English and Spanish.

| User | Language | Percentage |
|---|---|---|
| 1 | English | 76 |
| 2 | English | 78 |
| 3 | English | 83 |
| 4 | English | 82 |
| 5 | English | 83 |
| 6 | English | 72 |
| 7 | English | 78 |
| 8 | English | 72 |
| 9 | English | 73 |
| 10 | English | 86 |
| 11 | English | 73 |
| 12 | English | 77 |
| 13 | English | 73 |
| 14 | English | 72 |
| 15 | English | 61 |
| 16 | English | 75 |
| 17 | English | 73 |
| 18 | English | 79 |
| 19 | English | 72 |
| 20 | English | 81 |
| 21 | English | 78 |
| 22 | English | 79 |
| 1 | Spanish | 74 |
| 2 | Spanish | 71 |
| 3 | Spanish | 74 |
| 4 | Spanish | 69 |
| 5 | Spanish | 78 |
| 6 | Spanish | 75 |
| 7 | Spanish | 79 |
| 8 | Spanish | 73 |
| 9 | Spanish | 77 |
| 10 | Spanish | 70 |
| 11 | Spanish | 72 |
| 12 | Spanish | 75 |
| 13 | Spanish | 69 |
| 14 | Spanish | 67 |
| 15 | Spanish | 76 |
| 16 | Spanish | 73 |
| 17 | Spanish | 53 |
| 18 | Spanish | 75 |
| 19 | Spanish | 75 |
| 20 | Spanish | 78 |

| | | |
|---|---|---|
| 21 | Spanish | 73 |
| 22 | Spanish | 63 |
